# Exploring Monocytes-Macrophages in Immune Microenvironment of Glioblastoma for the Design of Novel Therapeutic Strategies

**DOI:** 10.3390/brainsci13040542

**Published:** 2023-03-24

**Authors:** Matías Daniel Caverzán, Lucía Beaugé, Paula Martina Oliveda, Bruno Cesca González, Eugenia Micaela Bühler, Luis Exequiel Ibarra

**Affiliations:** 1 Instituto de Investigaciones en Tecnologías Energéticas y Materiales Avanzados (IITEMA), Universidad Nacional de Rio Cuarto (UNRC) y Consejo Nacional de Investigaciones Científicas y Técnicas (CONICET), Río Cuarto X5800BIA, Argentina; 2 Departamento de Patología Animal, Facultad de Agronomía y Veterinaria, Universidad Nacional de Rio Cuarto, Rio Cuarto X5800BIA, Argentina; 3 Departamento de Biología Molecular, Facultad de Ciencias Exactas, Fisicoquímicas y Naturales, Universidad Nacional de Rio Cuarto, Rio Cuarto X5800BIA, Argentina; 4 Instituto de Biotecnología Ambiental y Salud (INBIAS), Universidad Nacional de Rio Cuarto (UNRC) y Consejo Nacional de Investigaciones Científicas y Técnicas (CONICET), Rio Cuarto X5800BIA, Argentina

**Keywords:** glioblastoma, macrophages, monocytes, tumor microenvironment, targeted therapy, cell-based therapy

## Abstract

Gliomas are primary malignant brain tumors. These tumors seem to be more and more frequent, not only because of a true increase in their incidence, but also due to the increase in life expectancy of the general population. Among gliomas, malignant gliomas and more specifically glioblastomas (GBM) are a challenge in their diagnosis and treatment. There are few effective therapies for these tumors, and patients with GBM fare poorly, even after aggressive surgery, chemotherapy, and radiation. Over the last decade, it is now appreciated that these tumors are composed of numerous distinct tumoral and non-tumoral cell populations, which could each influence the overall tumor biology and response to therapies. Monocytes have been proved to actively participate in tumor growth, giving rise to the support of tumor-associated macrophages (TAMs). In GBM, TAMs represent up to one half of the tumor mass cells, including both infiltrating macrophages and resident brain microglia. Infiltrating macrophages/monocytes constituted ~ 85% of the total TAM population, they have immune functions, and they can release a wide array of growth factors and cytokines in response to those factors produced by tumor and non-tumor cells from the tumor microenvironment (TME). A brief review of the literature shows that this cell population has been increasingly studied in GBM TME to understand its role in tumor progression and therapeutic resistance. Through the knowledge of its biology and protumoral function, the development of therapeutic strategies that employ their recruitment as well as the modulation of their immunological phenotype, and even the eradication of the cell population, can be harnessed for therapeutic benefit. This revision aims to summarize GBM TME and localization in tumor niches with special focus on TAM population, its origin and functions in tumor progression and resistance to conventional and experimental GBM treatments. Moreover, recent advances on the development of TAM cell targeting and new cellular therapeutic strategies based on monocyte/macrophages recruitment to eradicate GBM are discussed as complementary therapeutics.

## 1. Introduction

Brain and other nervous system cancers are among the most fatal cancers in several countries around the world [1,2,3]. In 2019, there were 347,992 global cases of brain and Central Nervous System (CNS) cancers, which showed a significant increase in its incidence (94.35%) from the period between 1990 to 2019 [4]. An estimated 251,329 people passed away from primary cancerous brain and central nervous system (CNS) tumors in 2020 [5]. Among brain tumors, malignant brain tumor incidence rates are slightly decreasing over the last decade; however, mortality rates increased in the same period of time [1]. Specifically, in the malignant brain tumor group, 5-year glioblastoma (GBM) survival only increased from 4% to 7% during the last years [1]. However, survival rates vary widely and depend on several factors, including the degree of malignancy and cellular and molecular distinctive features.

Over the years, the identification of distinct genetic and epigenetic profiles in various brain tumors has improved the classification of more than 100 cancerous diseases that can appear in this preferential location and allows the discovery of new diagnostic, prognostic, and predictive molecular biomarkers to improve the prediction of response to treatment and therapeutic outcome [6]. The classification of brain tumors has experienced numerous changes over the past half century. The World Health Organization (WHO) has played a key role in the effort to split malignancies according to clinical and histological profiles from the first classification launched in 1979 [7]. This increased complexity as reflected in the last classification in 2021 summarizes the current understanding of the clinical, histologic, and molecular features of CNS tumors and paves the way for further precision in tumor classification and a shift towards increased use of targeted therapeutics [8].

Among malignant gliomas, GBM is one of the most aggressive malignancies, accounting for 14.5% of all central nervous system tumors and 48.6% of malignant central nervous system tumors [9]. The median overall survival (OS) of GBM patients is only 15 months, which highlights the failure with conventional treatments applied so far [10]. The ongoing effort to identify potential new molecular or cellular targets for the development of effective clinical therapies has not yet led to significant improvements in survival rate, with most patients surviving not more than a few years. In this sense, the understanding of the molecular interactions among not only tumor cells but also other types of non-tumor cells that reside into tumors has made it possible to improve therapeutic targeting [11]. Nevertheless, the majority of studies related to GBM treatments over the last decades has focused on eradication of tumor cells, whereas more recent efforts have been placed on understanding the microenvironment surrounding tumor cells, the interaction between these cellular and acellular components in different preformed tumor niches, and how to design new treatment options that target these components in a multi-attack approach [12,13]. Tumor-associated macrophages (TAMs) play an essential role in the GBM microenvironment since this non-tumoral cell population represents up to 50% of tumor mass and specific treatments to eliminate these cells have been proposed in the past [14,15]. In this review, updated research on the components of the tumor microenvironment (TME) in GBM is presented, with a special focus on the main non-tumor cell population represented by macrophages and their location into GBM tumor niches. The main aspects included in the analysis are related to the origin of these cells, their recruitment within the GBM, their participation in the gliomagenesis process as well as in the resistance to the main treatments used. Moreover, the main findings related to the therapeutic targeting of macrophages based on their recruitment, polarization, and functions for GBM therapy are presented.

## 2. Search Strategy and Selection Criteria

The original published research studies in peer-reviewed journals cited in this review were published between 2014 and 2023, with a major focus on the years 2018 to 2022. The PubMed, Scopus, Google Academic, and the US National Institutes of Health Clinical Trials Registry (http://www.clinicaltrials.gov, accessed on 10 December 2022) databases were used to search relevant studies with the following keywords: “malignant gliomas”, “glioblastoma”, “tumor microenvironment”, “macrophages”, “targeting macrophages”, “microglia”, “monocyte recruitment” in different combinations. Duplicates and articles in languages other than English were excluded. Full articles with restricted access were also excluded. All references were cited to the content-related parts of the review.

## 3. Classification, Biological Features, and Tumor Niches of GBMs

Tumors generated from different glial cells in the CNS are known as gliomas. To unify the diagnostic criteria, WHO proposed a CNS tumor classification and nomenclature guide based on the combination of parameters such as tumor mass extension into the brain tissue, the proliferation of the microvasculature, genetic alterations, presence of necrotic areas, and cell proliferation index [16]. Low-grade gliomas (LGG) (grades 1 and 2) are less invasive while high-grade gliomas (3 and 4) represent the most challenging brain tumors. WHO Classification of Tumors of the CNS (WHO CNS5), revised recently, has suffered substantial changes by moving further to advance the role of molecular and genetic biomarkers’ identification in the diagnostics of CNS tumor classification but remaining rooted in other established approaches to tumor characterization, including histology and immunohistochemistry [8]. In addition, the number denoted in the gradation is now Arabic instead of a Roman numeral. This classification would have an impact in the correct diagnosis, treatment definition, and prognosis of the disease. For example, the identification of mutations in isocitrate dehydrogenase (IDH) defines gliomas with the best prognosis independently of their tumor grade [17]. IDH mutation in GBM is frequently associated with TP53 mutation, and it has a generally better prognosis than IDH-wildtype glioblastoma.

Among malignant gliomas, grade 4 tumors or GBM are the most aggressive, and they possess high levels of intratumoral and intertumoral heterogeneity. Apart from containing different genetic signatures, GBMs present different transcriptomic profiles, which have recently originated a new classification: classical, mesenchymal, neural, and proneural tumors [18]. However, this classification does not impose a different therapeutic approach, so it is not routinely performed in the clinic [11]. For this reason, the WHO classification includes GBM as part of the diffuse astrocytic and oligodendroglial tumors group and they are divided into three subgroups based on IDH mutations: (1) glioblastoma, IDH-wildtype, clinically identified as primary GBM and predominant in patients over 55 years of age, (2) glioblastoma, IDH-mutant, clinically identified as secondary GBM and more common in younger patients, and (3) glioblastoma NOS (not otherwise specified), which does not fit into the other categories and is not well defined [9].

During the gliomagenesis process, different genetic abnormalities signatures lead to GBM malignant cell transformation; however, tumors masses formed need a great amount of genetic, epigenetic, and metabolic changes in order to continue proliferation and expansion to the surrounding healthy brain tissue, including changes in energetic metabolism, invasive capacity, remodeling of the extracellular matrix (ECM), cell migration and promotion of angiogenesis [9]. The detachment of invading tumor cells from the primary tumor mass accompanied by decreased expression of Cx43 and increased CD44 expression, followed by the anchored and degradation of ECM by overexpressed MMP-9 and MMP-2, allow the colonization of tumor cells into normal brain tissues such as brain parenchyma, leptomeningeal space, white matter tracts of corpus callosum, and perivascular space [19,20]. GBM cells also attract non-tumoral cells such as microglia, astroytes, and endothelial cells that secrete proteases to enhance migration [14]. In this migration movement, tumor cells in immediate proximity of pre-existing and degenerated vessels begin to die, forming foci of necrosis. These foci become surrounded by tumor cells, which eventually form pseudopalisade and upregulate the expression of vascular endothelial growth factor (VEGF), leading to vascular hyperplasia, distinguishing glomeruloid vascular proliferation areas. Different niches within the tumor mass will be created, which contemplate the coexistence of tumor cells and non-tumor cells in different areas such as the hypoxic/necrotic niche, invasive front, and perivascular zones that not only define different cell constituents [21], but are also characterized by cell plasticity, heterogeneity, and resistance to radiotherapy and chemotherapy [12].

## 4. Tumor Microenvironment (TME) in GBM Niches

TME plays an essential role in cancer development. Various non-tumor cells participate in the TME, collaborating in growth, survival, invasion, and metastasis of tumor cells [22]. Tumor cells structure the tumor parenchyma and non-tumor cells, which are part of the stroma, have a cellular heterogeneity. Normal and reactive astrocytes, fibroblasts, immune cells, microglia, macrophages, endothelial cells, and vascular pericytes are part of the microenvironment of the GBM. Furthermore, proteins and non-protein biomolecules (polysaccharides, hormones, nitric oxide, etc.) are produced by all the cell types to promote neoplastic growth, and they are also main components of the TME [23]. More importantly, glioma stem cells (GSCs) have the capacity to generate new tumor cells and support cancer growth and regrowth even after the majority of treatments employed [22]. The location of GSCs into the tumor has been discussed, but they can be found in different niches of GBM close to central necrosis [22].

Perivascular niches are composed of blood vessels such as capillaries or arterioles, and GSCs have close contact with them [24]. Furthermore, reactive astrocytes presented in these areas generate angiopoietins 1 and 2 (Ang-1 or Ang-2) and VEGF, which are important cytokines for tumor cells that use the perivascular space for invasion and co-opt existing vessels as satellite tumors [25]. VEGF induced Ang-1 pericytes’ recruitment to improve vascular stability. Moreover, these molecules also participate in the recruitment of myeloid cell populations into GBM [26,27]. Around necrotic zone, Ang-1 is absent because hypoxia down-regulates Ang-1 expression; nevertheless, Ang-1 is more perceived in the tumor periphery [28].

The main molecular inductor of angiogenesis in perinecrotic areas is hypoxia-inducible factor 1 (HIF-1), which intensifies VEGF expression after translocation to nuclei [28]. On the other hand, perinecrotic niches are considered zones of high tumor cell proliferation and low endothelial cell development. An important feature in necrotic foci is the appearance of GSC around them [28].

Moreover, other non-cellular components belonging to ECM are upregulated into TME, such as hyaluronan, vitronectin, osteopontin, tenascin-C, SPARC, and BEHAB with an impact on the GBM progression. Their overexpression is correlated with poor prognosis [29]. This is of particular interest because hyaluronan helps in the progression of malignant gliomas by facilitating primary brain tumor invasion in and migration through its two cellular receptors, CD44 and RHAMM [29]. CD44 is the major receptor for hyaluronan and it contributes to cell–matrix interactions, cell migration, and regulation of tumor growth [29]. Tight junctions between ECM components and integrins of neoplastic cells lead to an increment in apoptotic resistance, proliferation, and migration [30]. Other overexpressed proteins such as fibronectin, which has the ability to regulate cell adhesion and migration, have been proposed as promoters of tumor invasion [31]. The overexpression of TGF-β, TGF-α, EGF, VEGF, and TNF-α promote both survival and tumor proliferation of GBM [32]. Many GBMs present EGFR amplification and/or mutation, and to a lesser extent they overexpress PDGF receptors. Those EGFR-dependent tumors would develop drug treatment resistance [33,34].

TAMs play an essential role in the GBM microenvironment. These cells can come from two different tissue origins. Microglia cells are derived from primitive hematopoiesis in the fetal yolk sac and take up residence in the brain during early fetal development [35]. Microglia differentiation and proliferation requires colony-stimulating factor 1 (CSF1), CD34, and the transcription factor PU.1 [35]. Under normal physiological conditions, the brain is only occupied by resident microglia, and the presence of other bone-marrow-derived macrophages (BMDM) are associated with the diseased brain. Microglia are long-lived and have self-renewal capacity compared with BMDM [36]. In addition, peripheral macrophages driven by inflammatory factors from GBM tumor cells and other TME cell populations promote the infiltration of circulating BMDM derived from hematopoietic stem cells that can migrate to tumor tissue; they penetrate the blood–brain tumor barrier (BBTB), and probably the intact blood–brain barrier (BBB), where they differentiate into monocyte-derived macrophages and promote tumor progression [14,37]. The BBB provides both a physical and a physiological barrier between the brain parenchyma and the bloodstream restricting the entry of various components such as peptides and proteins, due to tight junctions [38] and also limits the permeability of immune cells from blood [39]. Upon brain injury produced by GBM tumorigenesis, the BBB becomes compromised (forming the BTBB) leading to significant influx of circulating BMDM and other immunological cells [40]. Moreover, Wang L.J. reported through immune landscape analysis that the risk score was significantly related to TME, specifically taking into account the macrophage cell population in malignant gliomas. Authors demonstrated the value of TAMs-related signature in predicting the prognosis of glioma, and they provided potential targeted therapy for glioma by in silico analysis [41]. Pinto L. et al. analyzed and characterized myeloid and lymphoid infiltrate in grade 2, 3, and 4 gliomas human samples by multicolor flow cytometry, along with the composition of the cell subsets of circulating myeloid cells [40]. They described that the infiltration by BMDM reached the highest percentages in GBM, and it increased from the periphery to the center of the lesion, where it exerted a strong immunosuppression that was absent in marginal areas instead. Chen et al. in 2017 agreed that BMDMs predominate within the GBM parenchyma, while microglia reside at the tumor periphery, so TAMs are represented by ~85% of infiltrating BMDM and ~15% of microglia [15].

**Figure 1:**
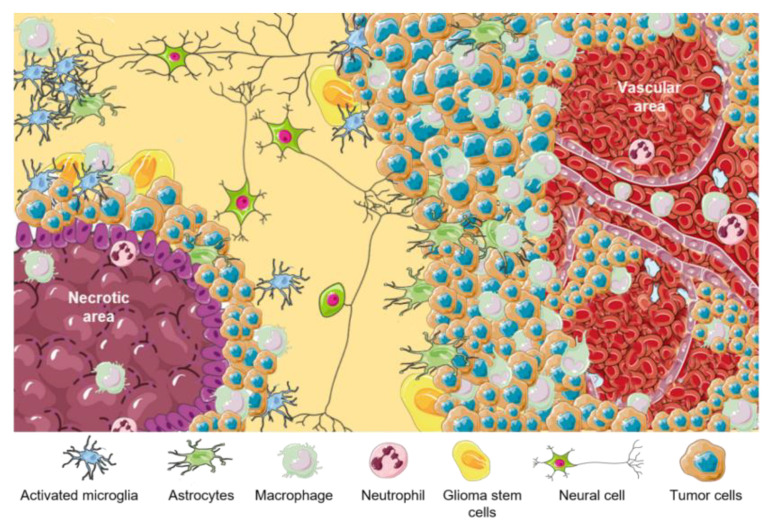
TME in GBM. Representative scheme of different GBM tumor areas. TAMs are associated with perinecrotic core centers, perivascular areas, and tumor front invasion zones. This figure was created using Servier Medical Art templates, which are licensed under a Creative Commons Attribution 3.0 Unported License; https://smart.servier.com (accessed on 1 February 2023).

Thus, the majority of immune cells in GBM includes a vast diversity of myeloid and lymphoid cells, which comprise BMDMs, myeloid-derived suppressor cells (MDSCs), DCs, lymphocytes, natural killer (NK), neutrophils, etc. [42]. However, the complex cell–cell interactions provide a unique physiological advantage for glioma cells that establishes an immune-suppressive and tumor-development-permissive microenvironment that is featured with high resident and recruited myeloid cell substances, hyporesponsive, and exhausted tumor infiltrating lymphocyte (TIL), which makes malignant glioma known as an immunologically “cold” tumor [43,44]. In addition, some studies indicated that reducing the number of MDSCs recruitment may slow the progression of glioma tumor cells [45]. Lymphoid cells are presented in GBM, but they are infrequent and they represent less than 2% of the tumor mass [46]. A representative scheme of different cell components of GBM TME is summarized in Figure 1. Principal functions of GBM cellular components are listed in Table 1.

As we mentioned previously, the prominent genomic feature that mostly distinguishes LGG from malignant gliomas, such as GBM, is the mutational status of the two genes encoding the cytoplasmatic IDH1 and/or the mitochondrial IDH2, where ~80% of LGGs present IDH mutations, compared to only ~5% of GBMs. Interestingly, IDH mutations are an independent prognostic factor in gliomas and they are associated with increased survival in all types, including GBM [17,47]. IDH status also denote TME cell components differences between tumors with the wild-type isoform and those with the mutated IDH [48]. Unlike GBMs with IDH-wildtype, GBMs with the IDH mutation have been shown to have less M2 macrophage infiltration and fewer PD-1-expressing T cells [49]. A study based on samples from patients with GBM showed that there is less infiltration of TAMs in GBM with IDH mutation, being more proinflammatory, which could reflect a better prognosis for these patients, and the fact that microglia in mutated IDH also have a proinflammatory role [50].

## 5. Monocyte Recruitment as Main Source of TAMs in GBM

It is well-known that numerous types of circulating cells are recruited into tumor tissues. After migration from the bone marrow into the peripheral blood, monocytes enter different tissues, and they differentiate into macrophages. There is increasing evidence that monocytes, in particular, migrate into GBM, where they differentiate into macrophages and they accumulate in distinct zones of the TME depending on the pattern of chemokine expression and secretion [51].

It has not been long since it has been recognized that TAMs from GBMs have a monocyte origin besides microglial origin and that the recruitment of different types of monocytes from the bloodstream is closely related to the GBM microenvironment and its different areas, and the BBB does not necessarily have to be disrupted [52,53]. Monocytes are not a homogeneous population, but they rather vary in phenotype and function. Based on this, monocytes from mice can be divided into two main subsets based on the expression of *LY6C* and *CX3CR1* genes which have been termed classical and non-classical monocytes [15,54]. Human monocytes are commonly divided into three subsets based on CD14 and CD16 expression, and the recent incorporation of 6-sulfo LacNac (SLAN) expression allows a better differentiation between subtypes [53]: classical monocytes (CD14+ CD16− SLAN−), intermediate monocytes (CD14+ CD16+ SLAN−), and non-classical monocytes (CD14low/− CD16+ SLAN+) [55]. Classical monocytes, similar to those of mouse LY6C^HI^ monocytes, highly express CCR2; they are the most prevalent monocyte subset in human blood, and they are recruited in inflamed environments [52].

**Figure 2:**
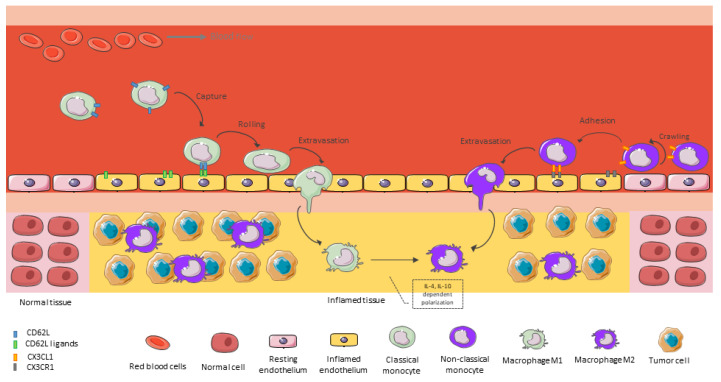
BMDM recruitment into inflamed brain tissue. An activated endothelium allows the recruitment of cells from the bloodstream through a well-orchestrated and coordinated mechanism. This figure was created using Servier Medical Art templates, which are licensed under a Creative Commons Attribution 3.0 Unported License; https://smart.servier.com (accessed on 1 February 2023).

As previously mentioned, when monocytes extravasate and reach the GBM tumor mass, they begin to differentiate into mature macrophages. In this step, tumor-derived chemokines and monocyte chemokine receptors play a critical role in monocyte/macrophage recruitment (Figure 2). Over the last century, it has been shown that various receptor–ligand pairs can regulate monocyte/macrophage recruitment into specific tumor microenvironments. Among the receptor-ligand pairs, the ligands of CD62L/CD62L, CCR2/CCL2, CX3CR1/CX3CL1, and VEGFR1/VEGF-A have been the most significantly implicated in monocyte/macrophage recruitment into specific TME areas. Ligands for these receptors are produced in the TME by GBM tumor cells, leukocytes, endothelial cells, and infiltrating fibroblasts, and their expression has been shown to positively correlate with the number of macrophages in tumors [15,56,57].

CCL2, also known as monocyte chemotactic protein-1 (MCP-1) or small inducible cytokine A2 (SCYA2), is a highly potent chemoattractant of monocytes/macrophages to areas of tissue injury and inflammation, as well as to tumor areas. Many studies have made it clear that CCL2 is the primary cytokine in monocyte recruitment into the inflamed CNS [40,53,58,59]. Moreover, the extent of CCL2 expression is associated with glioma grade [60]. In the setting of murine GBM, research has shown that neoplastic cells in GBM express high levels of CCL2, which contributes to the directional infiltration of CCR2^Hi^ inflammatory monocytes into the tumor [61]. CCL7 also mediates the recruitment of BMDMs via binding to CCR2 [62]. Loss of CCL2 or CCL7 can significantly reduce the recruitment of BMDMs (40–50% reduction) during inflammation processes and it enhances therapeutic response [63]. Additionally, it was shown with orthotopic GSC xenografts that periostin secreted by tumor cells specifically supported the recruitment of anti-inflammatory and consequently pro-tumor monocyte-derived macrophages, a result validated with immunohistochemistry on human GBM tissue, which showed more CCR2+ cells in the tumor infiltrate [64].

GBM tumor niches could recruit different subtypes of monocytes. For instance, due to reduced oxygen supply, the central regions of GBM tumors show high levels of hypoxia and in this hypoxic region, hypoxia-inducible chemokines that attract monocytes/macrophages, such as VEGF-A, SDF-1 are enriched compared to the peritumoral region [28,65,66,67]. On the other hand, CX3CL1/CX3CR1 chemokine axis elicited adhesion and migration of TAMs, they increased the expression of matrix metalloproteinase (MMP) 2, MMP9, and MMP14 enzymes that degrade ECM, and they are concerned in tumor invasiveness [68]. For this reason, this axis is more implicated in the non-classical monocyte recruitment into the perivascular area. CX3CR1 signaling enhances accumulation of BMDMs and angiogenesis during malignant transformation of LGG [69]. In another study, the expression of CX3CL1 was inversely correlated with patient overall survival with the uppermost scores of CX3CL1 expression in grades 3–4 tumors: oligodendrogliomas, anaplastic astrocytomas, and GBM [70]. In concordance, a recent study demonstrated that the transcripts of seven chemokines, including CCL2, CCL8, CCL18, CCL28, CXCL1, CXCL5, and CXCL13 were highly expressed in GBM, which was also evidenced with a large immune cell infiltrate and it was accompanied by worse GBM patient outcomes [71]. CCR2^Hi^ inflammatory monocytes are rapidly recruited to sites of inflammation and sites of tissue remodeling as well, and they have been shown to be the major source of TAMs in GBM [15,72]. These monocytes will be homed in perivascular and perinecrotic/hypoxic areas [72]. For instance, Chen et al. demonstrated that CCR2+ inflammatory monocytes are rapidly recruited into a GBM orthotopic mouse model and they are highly motile cells to reach different zones, but they also could rapidly change to a stationary CX3CR1^hi^CCR2^lo^ and CX3CR1^hi^CCR2– TAM profile in perivascular areas adjacent to endothelial cells and pericytes [73].

Another important chemoattractant axis for TAM recruitment is CXCL12/CXCR4 axis. As it was previously mentioned, CXCL12, also known as SDF-1 is enriched in hypoxic areas and it is related to glioma progression, cancer cell–TME interaction, cellular invasion, and tumor angiogenesis [67,74,75]. Angiogenesis is one of the key hallmarks of GBM, and CXCL12 binding to CXCR4 participates in this process via boosting VEGF release [67,76]. It has been reported that high CXCL12 levels in GBM may attract CXCR4-positive vascular and inflammatory cells such as TAMs that, once within the tumor, secrete tumor-promoting cytokines as well as growth and pro-angiogenic factors [77,78]. CXCR4 high levels of expression have been related to negative prognostic significance in malignant glioma patients [79]. Some of these ligand–receptor axes will be discussed later as targets to decrease TAM recruitment.

## 6. Macrophages Functions in Malignant Gliomas

Macrophages have been classified as M1 and M2 subtypes. These immune cells have clout in tumors due to M1 having better prognosis in patients than the infiltrating of M2 [80]. Macrophage subtypes have many differences, M1 cells have a proinflammatory phenotype that generate interleukin-1 (IL-1), IL-12, IL-23, IL-6, Tumor Necrosis Factor ɑ (TNF-ɑ), and ROS. In counterpart, M2 TAMs have an anti-inflammatory and tumor progression promoter phenotype, they generate IL-10, IL-4, IL5, VEGF, and they cause immune suppression promoting transforming growth factor β (TGF-β). Additionally, M2 helps recruit Th2 helper T cells, which release IL-4, IL-5, and IL-10 [81]. On the other hand, TAMs with an M2-like phenotype participate in the proliferation, survival, and migration of tumor cells [82]. It is known that TAMs release IL-6 and IL-1β that activate various cell proliferation pathways [83]. IL-6 secretion by macrophages is highly correlated with the poor prognosis of GBM patients, and its quantification in the cerebrospinal fluid was proposed as a prognostic marker [84].

In GBM, there is a predisposition for BMDMs to be found in the tumor nucleus in a greater proportion; however, microglia-derived TAMs are found in the periphery of the tumor [15]. In this regard, a study demonstrated that M2-like TAMs represented by macrophages CD204+ were correlated with poor prognosis in GBM and they expressed markers from both M1 and M2 activation profiles. Furthermore, these TAMs were located around blood vessels and perinecrotic areas, where a protumoral interaction with GSCs is postulated [85]. Perivascular TAMs (with a more M2 phenotype) are proangiogenic and protumoral, because they present a variety of markers such as VEGFA, CCR2, and Tie2 [86]. It has been reported that microglia/macrophages cells present proangiogenic factors such as CXCL2 and CD13 that act independently of VEGF. This could explain the recurrence of GBM and the failure of antiangiogenic therapies against VEGF [87].

Although the GBM TME exhibits proangiogenic characteristics through VEGF and other molecules, it is also characterized by the secretion of TGF-β by TAMs that acts by suppressing the function and proliferation of cytotoxic T cells [88]. Moreover, an important lymphocyte depletion is initiated in GBM due to the large presence of macrophages, with a suppressed Th1 profile and a higher M2 response [89]. The immunosuppressive effects of GBM can be attributed to the elevated levels of TGF-β, since it promotes the stimulation of the M2 phenotype in macrophages with release of the immunosuppressive cytokine IL-10. In addition, TGF-β decreases the production of molecules such as granzyme A/B, interferon gamma, and perforin, which are fundamental molecules in cytotoxicity mediated by NK and T cells [90]. Moreover, M2 macrophages express chemokines that increase the recruitment of regulatory T cells (Tregs), such as chemokine C-C ligand 2 (CCL2), CCL5, CCL20, and CCL22. These chemokines also inhibit the activity of CD4+ and CD8+ effector cells, NK cells, and DCs [40,91].

**Figure 3:**
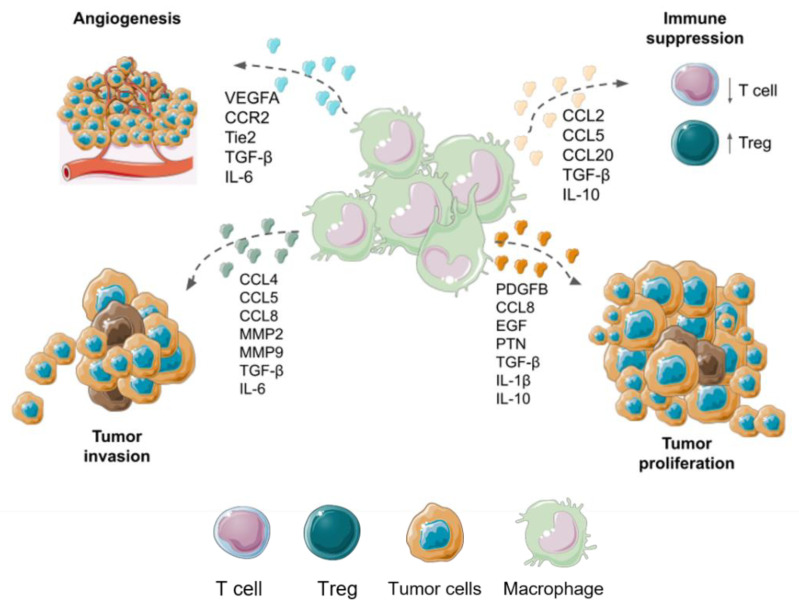
Macrophage functions supporting GBM malignancy. The role of TAMs in different biological events such as angiogenesis, proliferation, invasion, and immune suppression. This figure was created using Servier Medical Art templates, which are licensed under a Creative Commons Attribution 3.0 Unported License; https://smart.servier.com (accessed on 1 February 2023).

Microglia cells promote the invasion of neoplastic cells through the secretion of TGF-β, which promotes the release of MMP2 that degrades components of the ECM, such as gelatins, collagen, and elastin [31]. Additionally, TAMs release other invasion-promoting molecules such as CCL5 and CCL8, which degrade the ECM [92]. CCL5 of microglia/macrophages favors glioma tumor progression through the CC5 receptor (CCR5), therefore GBM patients who overexpress CCR5 have a worse prognosis. CCL5/CCR5 interaction triggers MMP invasion and intracellular calcium cascade [93]. Together with MMPs, ADAM (A Disintegrin and Metalloprotease) metalloendopeptidases are related to the progression of GBM. ADAM8 is expressed in both M1 and M2 macrophages, while MMP9 and MMP14 are associated with M2 and related with poor patient prognosis. MMP14 inhibition improved survival in experimental animals with GBM, and may be a possible therapeutic target [94]. On the other hand, the M1 phenotype of macrophages is allied with the expression of ADAM10 and ADAM17, resulting in a better prognosis for patients with GBM [95]. Different protumoral functions with principal molecules involved with microglia and BMDMs TAMs are summarized in Figure 3.

## 7. Conventional and Alternative Treatment Modalities for GBM

The current standard of care coordinates patients with newly diagnosed GBM to be treated with maximal safe resection surgery, followed by a course of radiotherapy (RT) with a simultaneous dose of temozolomide (TMZ), and then adjuvant chemotherapy of several maintenance cycles with TMZ (Stupp protocol). Post-surgery, the treatment regimen consists of 6 weeks of RT to the surgical cavity, followed by adjuvant chemotherapy, consisting of a total of six cycles of treatment with TMZ at a dose of 150–200 mg/m^2^ for 5 days for every 28-day cycle [10,96]. After this standard first-line treatment, the progression of the disease is highly heterogeneous with a median survival of 14.6 months, with only a 10% to 15% of patients reaching 3 years of life during the current standard-of-care period [97]. According to a systematic review of randomized clinical trials, RT plus TMZ provides better survival outcomes than RT alone [98]. However, long-term administration of TMZ generally generates resistance, limiting its efficacy. The contribution of macrophages to the therapeutic resistance of TMZ was also reported [99].

New therapeutic schemes include tumor-treating fields (TTFields) with low-intensity, alternating electric fields delivered by transducer arrays applied to the scalp over the regions of the brain where tumors are localized. The use of TTFields produces mitosis inhibition and cell cycle arrest, disturbs DNA repair, interrupts cell migration, and thus suppresses tumor growth and invasion [100,101]. The effectiveness and safety of TTFields in GBMs management have been confirmed in various randomized clinical studies, and it has been established as the fourth treatment option in addition to surgery, RT, and chemotherapy [102]. Nevertheless, TTFields given during maintenance TMZ still fails to improve the median overall survival (OS) for more than 21 months [13]. However, a benefit is the promotion of the production of immune-stimulating proinflammatory environment with recruitment of proinflammatory cells from blood such as monocytes [103].

Molecular targeting approach is another therapeutic strategy greatly explored in GBM. Most molecular therapies have been developed to specifically inhibit tumor angiogenesis [104,105] or to block ligand-independent and dependent signaling pathways, such as dual-targeted of PI3K/mTOR signaling with PDGFR and VEGFR inhibitors [57,106].

**Figure 4:**
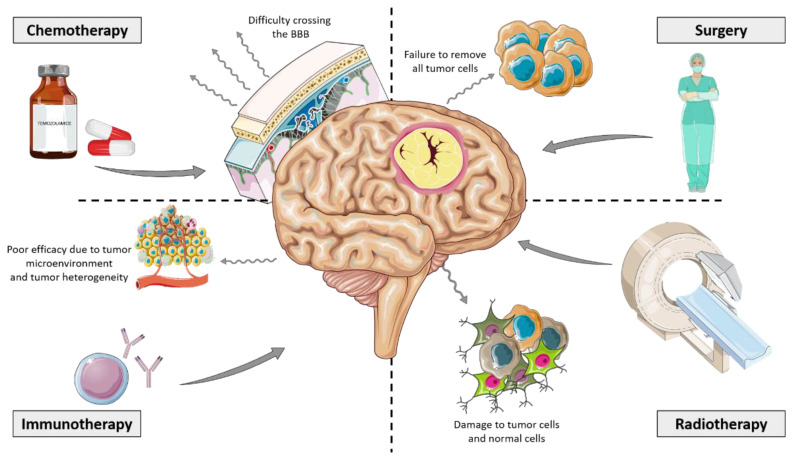
Conventional treatment modalities for GBM. Main treatment options for GBM patients are represented by solid straight lines. Limitations for these main treatments are schematized as refractory lines. This figure was created using Servier Medical Art templates, which are licensed under a Creative Commons Attribution 3.0 Unported License; https://smart.servier.com (accessed on 1 February 2023).

From an immunotherapy approach, treatments with immune checkpoint inhibitors such as anti-CTLA-4 mAb, PD-1 and PD-L1 inhibitors demonstrated improved OS in some patients with malignant gliomas, suggesting that immunotherapy is a potential treatment option for CNS tumors, mainly in combination modalities [107]. Despite this, a persistent challenge remains for immunotherapy in the treatment of GBM due to the existence of redundant mechanisms of tumor-mediated immune suppression from its environment. Dendritic cell (DC) immunotherapy is an alternative emerging strategy for the treatment of GBM. Recently, phase I and II clinical trials testing DC vaccines in patients with newly diagnosed and recurrent GBM were conducted. The results demonstrated that DC immunotherapy enhanced progression-free survival (PFS) in GBM patients and elevated numbers of tumor-infiltrating CD8+ lymphocytes [108]. Accordingly, Iurlaro R. and colleagues recently engineered T-cell bispecific antibodies (TCB) that bind both the T-cell receptor and tumor-specific antigens [109]. The tumor-specific antigen proposed by the group was the epidermal growth factor receptor variant III (EGFRvIII), which is expressed on the surface of tumor cells; it is not expressed in normal tissues, and it represents a common mutation event in GBM patients. EGFRvIII-TCB showed specificity for EGFRvIII and promoted tumor cell killing as well as T-cell activation. In addition, EGFRvIII-TCB promoted T-cell recruitment into GBM animal models [109]. Advantages and limitations for conventional treatments are shown in Figure 4.

**Figure 5:**
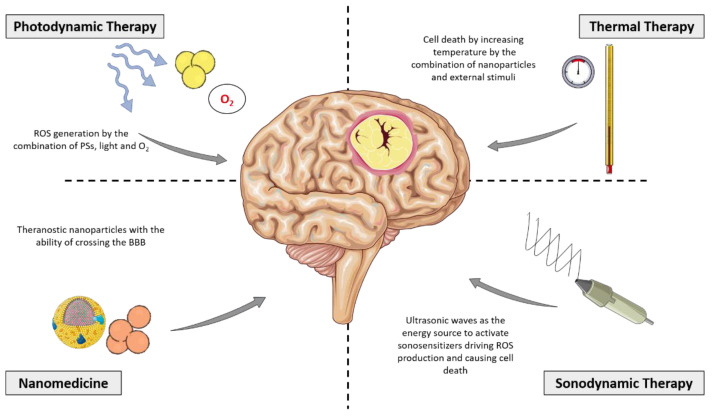
Alternative treatment modalities for GBM. Non-standard treatment options for GBM patients with the main mechanisms of action are represented by solid straight lines. This figure was created using Servier Medical Art templates, which are licensed under a Creative Commons Attribution 3.0 Unported License; https://smart.servier.com (accessed on 1 February 2023).

Alternative treatment modalities such as photo-assisted therapies have extensively been validated for newly diagnosed and recurrent GBM [110,111,112]. When glioma cells absorb a molecule called photosensitizer (PS), exposure to high intensity laser light will be able to kill tumor cells by light activable reactive oxygen species (ROS) reactions in the photodynamic therapy (PDT) [113,114]. Clinical trials with classical PS have been conducted in a few countries such as Australia, France, and Japan, where results in newly diagnosed HGG patients indicate greater success (NCT01966809, NCT01148966, NCT04391062, JMA-IIA00026) [115,116]. PDT approach not only involves direct tumor cell destruction, but also the mechanisms of ROS-mediated activation can promote other antitumoral effects such as the activation of immune response [117], a vascular supply reduction [118], and also the opening of the BBB to enhance drug permeability into brain tissue [111]. Photoactivation of PSs also allows the emission of fluorescence and phosphorescence that can be used in the diagnosis of remaining tumor cells and/or delimitation of surgical margins [119,120]. A challenge for some photo-assisted therapies is the requirement of all the elements needed in the tumor site. In this sense, devices to activate sensitizers are not found everywhere. Alternative treatment modalities in preclinical and clinical trials are shown in Figure 5. Other limitations for these new therapies come from TME such as the presence of endothelial cells of the BBB, macrophages engulfing therapeutic nanoparticles, hypoxia developed by tumor growth, etc. [121]. PDT and sonodynamic therapy (SDT) need the consumption of oxygen to generate ROS and induce cancer cell death. Under a hypoxia environment, the reduced oxygen supply is a challenge for both PDT and SDT. However, TME components could offer therapeutic strategies that can be applied with nanotechnology to achieve higher specificity for target cells and avoid damage to nearby healthy tissue. For instance, nanoparticle surfaces have been functionalized with various targeting moieties for molecular recognition of tumoral and non-tumoral cells [122]. In another approach, nanoparticles have been developed to employ tumor hypoxia or oxidative stress to accomplish a therapeutic effect [121].

As it can be seen, the therapeutic approach for GBM requires a multiple attack towards several molecular targets with the help of surrounding cells. This highlights the importance of studying the intercellular relationships between tumor cells and other types of non-tumor cells inhabiting the tumor mass. From this point of view, the principal non-tumoral cell population represented by TAMs could help to improve the efficacy of different treatments modalities. In the following section, a brief examination of treatments that focus on TAMs and that can be used in combination with the above treatments will be discussed.

## 8. Therapeutic Strategies Focused on TAMs of GBM

The overwhelming evidence of the presence of TAMs in the immune infiltrate of both murine and human malignant gliomas has raised awareness of the persuasive role these cells may have on several biological events to develop an immunosuppressive environment, enabling the glioma cell progression and invasion and the contribution to the resistance to many treatment interventions. Understanding the phenotype, function, and the cell programming or plasticity of these cells is of great importance since the focus on glioma therapy is shifted towards targeting the microenvironment cells as well as the tumor cells.

### 8.1. Strategies to Deplete Macrophages or Inhibit Monocyte Recruitment into GBMs

Macrophages of malignant glioma TME are characterized by their plasticity and heterogeneity; however, in a dichotomy approach where two extreme types of macrophage phenotypes co-exist in gliomas’ TME, pro-tumoral M2 macrophages with low expression of IL-12, IL-23, and a high expression of IL-10 and TGF-β have become an attractive therapeutic target to help eradicate this type of tumor. Furthermore, M2 macrophages also have high levels of arginase 1, mannose receptors, and scavenger receptors that serve to classify these cells in the context of several tumors. Studies have shown that TAMs are highly implicated in suppressing anti-tumor immune functions of T cells and directly facilitate tumor cell immune escape [123]. For these reasons, as the higher macrophage infiltration in the TME of GBM is often correlated with poor treatment outcomes and prognosis [27], depleting them by specifically targeting and killing them is an attractive strategy that was evaluated in the recent past.

One of main strategies to deplete TAMs is to target the colony-stimulating factor 1 receptor (CSF-1R). CSF-1R belongs to a type III protein kinase receptor family and binds to two ligands, CSF-1 and IL-34. After binding, ligands induce homodimerization of CSF-1R and activation of receptor signaling, which is crucial for the differentiation and survival of macrophages in tissues [124]. Gabrusiewicz K. et al. modeled in vivo GBM using intracranial GL261-bearing CSF-1R–GFP+ macrophage Fas-induced apoptosis transgenic mice [125]. In their mice, transitory macrophage population ablation was achieved by exposure to AP20187, a ligand which induces Fas-mediated apoptosis through activation of the caspase-8 pathway in myeloid lineage cells; and afterward, tumors showed lower mitotic index, microvascular density, and a reduction in tumor growth [124]. In order to achieve depletion of TAMs by CSF-1R, small molecule inhibitors and monoclonal antibodies were developed in the last decade with a few of them reaching clinical trials for the GBM treatment as monotherapy or in combination with other drugs [126,127]. For instance, cabiralizumab, a recombinant monoclonal antibody to CSF-1R, is in a phase 1a/1b doses-escalation study alone and in combination with nivolumab, another monoclonal antibody anti-PD-1 for advanced solid tumors including malignant gliomas (NCT02526017). An example of a small molecule against CSF-1R is PLX3397, a potent CSF-1R and c-Kit inhibitor [128], which is also in clinical trials for recurrent GBMs (NCT01349036, phase 2 study—terminated) and also for newly diagnosed GBMs in combination with TMZ and RT (NCT01790503). These drugs demonstrated outstanding results in preclinical mice models [128,129]; however, their efficacy in human GBM is still under investigation.

Another strategy to deplete TAMs, which is currently under evaluation is to target CXCR4 with antagonists. CXCR4 is overexpressed in numerous human cancers including glioma, and it has been shown to promote tumor growth, invasion, angiogenesis, metastasis, relapse, and therapeutic resistance [56]. In addition to being overexpressed in tumor cells and GSCs, it is also found in TAMs. AMD3100, USL311, and POL5551 have been used to deplete TAMs in GBM in combination with chemotherapy, RT, and antivascular therapy [65,130,131]. Gagner J.P. et al. demonstrated that the combination of POL5551 and B20-4.1.1, an anti-VEGF antibody, reduced tumor invasiveness, vascular density, and reduced Iba1-positive microglia TAM population within tumors compared to antivascular therapy alone in preclinical GBM mouse models [65]. It is known that the action of antiangiogenic agents in malignant gliomas is not very effective and leads to a greater accumulation of immunosuppressive myeloid populations in hypoxic areas [132]. Their findings raise the possibility that CXCR4 antagonists may interfere with the microglial mechanism of escape of GBM to anti-VEGF therapy. A clinical trial, which has already ended, evaluated USL311 as a single agent and in combination with lomustine for advanced and recurrent GBM through a phase ½ dose-escalation study in order to determine treatment modality and regimen of administration (NCT02765165). A common feature among these therapies is that they are ineffective when applied alone and require a combinatory modality to succeed.

### 8.2. Strategies to Reprogramme TAMs to an Antitumoral and Phagocitic Profile

Although TAMs with a M2-like phenotype play an important role in tumor development and progression, M1 TAMs have been shown to effectively eliminate cancer cells [133,134]. Reprograming TAMs from their tumor supporting phenotype (M2) towards an anti-tumor phenotype (M1) can therefore inhibit tumor growth and enhance an anti-cancer immune signaling. To achieve this, several molecules have been reported in TAM or in glioma cells from which molecular interactions with TAM perpetuate the M2 phenotype and could be therapeutic targets. Experimental studies have revealed a macrophage-mediated drug resistance mechanism in which the TME undergoes adaptation in response to macrophage-targeted CSF1R inhibition therapy in gliomas. As we previously mentioned, CSF1R targeting not only diminishes TAM population but also its blockage could revert polarization to a M1 phenotype [129]. Sun et al. demonstrated how macrophage phenotype could be exploited to exert anti-tumor effects by treating macrophages with an inhibitor of the CSF1R, thus making them switch from M2 to M1 phenotype and stimulating phagocytosis of tumor cells. However, after prolonged treatment with CSF1R inhibitors, IL4 accumulated from other TME cell types stimulated TAMs to secrete insulin-like growth factor 1 (IGF1), which in turn sustains the survival and growth of glioma cells [135]. For this reason, a combined treatment modality with CSFR inhibition and IGF1 receptor (IGF1R) inhibition will be the goal of designing more effective therapies for gliomas [135]. In another approach to reprogram TAMs, Mukherjee S. et al. developed novel liposomal formulation of TriCurin (TrLp). TriCurin is a mixed of curcumin with two other polyphenols, epicatechin gallate from green tea and resveratrol from red grapes. These TrLp liposomes were able to produce a major stimulation of the innate immune system by repolarizing TAM to the tumoricidal M1-like phenotype and also triggering intra-tumor recruitment of NK cells from the bloodstream into GBM GL261 mouse model [136].

Toll-like receptors (TLRs) are essential in the recognition of molecular patterns enhanced by a broad spectrum of infectious agents, and they stimulate a variety of inflammatory responses. Among them, TLR9 is expressed intracellularly in innate immune cells within the endosomal compartments, and it is activated by its binding to DNA rich in CpG motifs. A recent study has shown that fungal polymer Schizophyllan (SPG)-based nanoparticles (well-known ligand for Dectin-1 receptors) entrapping short DNA CpG ODN 1826 activated the signal transducer and activator of transcription 1 (STAT1) within GBM TAMs, which in turn promotes the synthesis of Th1-type cytokines such as IL-1β, IFN-γ, iNOS, and TNF-α and further restricts tumor growth [137]. Another receptor evaluated to reprogram M2 TAMs was H1 histamine receptor (Hrh1), which is significantly upregulated in the M2-like compared M1-like TAMs. Chryplewicz et al. demonstrated that imipramine, a re-purposed tricyclic antidepressant reprogrammed TAMs into a pro-inflammatory M1 phenotype, and these cells were responsible for the recruitment of T cells, in part by expressing the chemokines CXCL9 and CXCL10 [138].

As well as the activation of STAT-1 is associated with the transcription of genes related with the M1 profile in TAMs, the activation of STAT-3 has an opposite effect, activating genes related to anti-inflammatory proteins and therefore polarizing macrophages towards an M2 profile. STAT3 is a cytoplasmic transcription factor that regulates cell proliferation, differentiation, apoptosis, angiogenesis, inflammation, and immune responses [139]. Aberrant STAT3 activation triggers tumor progression through oncogenic gene expression in numerous cancer types including malignant gliomas [140,141]. Moreover, STAT3 activation in immune cells causes elevation of immunosuppressive factors [142]. One of the first studies that validated STAT3 inhibition as a repolarization strategy towards an M1 profile in GBM TAMs with a beneficial outcome was presented by Zhang L. et al. in 2009 [143]. In this study, CPA-7, an inhibitor of Stat3 dimerization, and STAT3 siRNA were used efficiently to reverse the immune profile of TAMs and cause tumor growth inhibition in the GL261 GBM mouse model [143]. The effect of TAM with active STAT3 leads to the secretion of interleukin (IL)-1β, which promote GBM growth by also allowing the activation of STAT3 and nuclear factor-kappa B (NF-kB) signaling in tumor cells [144]. In a recent study, STAT3 activation in GBM cells stimulated by TGF-β and released by M2 TAMs allows GSCs maintenance and self-renewal as a main tumor growth mechanism [145]. Furthermore, noncoding RNAs were postulated to play an important role in upstream signals to regulate the expression and activation of STAT3 in TME cells [146]. For example, it was proposed that miR-1246, derived from hypoxic glioma cells, induced M2 TAM polarization by targeting TERF2IP to activate the STAT3 signaling pathway [147]. Targeting this microRNA may contribute to antitumor immunotherapy in GBM patients.

CD40 is expressed on several antigen presenting cells including TAMs. CD40 has been proposed as a molecular target to reprogram M2 TAMs to an antitumoral phenotype in GBM management. In order to accomplish this, agonistic CD40 monoclonal antibody (mAb) has been used [148,149]. In fact, some studies have shown efficacy combining these mAB with other molecular treatments to increase therapeutic success, such as COX-2 and IL-6 inhibitions [150,151]. Therefore, re-education of TAMs rather than depletion may represent a more effective strategy as monotherapy or in a combination modality.

### 8.3. Cell-Based Therapy Using Monocytes-Macrophages for GBM

Taking into account that mononuclear phagocytes are in constant traffic into tumors, macrophages have been explored on their own as therapeutic agents in the TME of different type of cancers [37,152,153]. In the new era, the use of biological agents as medicinal products is revolutionizing the field of medicine. These products are obtained from living organisms or their tissues, which include viruses, serum, toxins, antitoxins, vaccines, blood components or derivatives, allergenic products, hormones, cytokines, antibodies, among others. Somatic cell therapy involves the use of cells collected from patients that must have undergone “more than minimal manipulation” (propagation, expansion, selection, or pharmacologically treated to alter the biological characteristics of the naïve cells) to accomplish a therapeutic action. In this sense, monocytes–macrophages could serve as advanced therapy medicinal products (ATMPs) and they are been explored for several diseases [153,154,155].

Strategies for macrophage cell therapy are based on the fact that monocytes are capable to act as Trojan horses, delivering small molecules such as cytokines, miRNA [156,157], or nanoparticles to the TME [158,159], and it is also possible to modify these cells with engineered receptors to achieve a better homing performance into tumors [160]. In concordance, cell delivery with other cells such as mesenchymal stem cells, monocytes, and neutrophils has been also used in the targeted delivery of a wide variety of anticancer agents, including nanoparticles, chemotherapeutics, proteins, suicide genes, and viruses [161,162,163,164]. Unlike other anticancer agents, these cells migrate to and infiltrate tumors through an active process despite high interstitial pressures and stromal barriers. This “tumor-homing” capacity is achieved through cytokine gradients, growth factors, ECM remodeling enzymes, and chemokines [37]. Recently, monocytes have been used as carriers of conjugated polymer nanoparticle for improving PDT management of GBM in vitro e in vivo [161]. In this study, inflammatory activated monocytes engulfed huge amounts of nanoparticles without affecting cell viability or chemotactic ability towards GBM orthotopic tumors. In addition, circulating monocyte-derived macrophages loaded with phototherapeutic nanoparticles were able to penetrate deeper GBM spheroids by increasing the spatial distribution of the nanoparticles in these three-dimensional models achieving an improved PDT outcome [161]. Another study used primary M1 macrophages as multifunctional carriers combined with PLGA nanoparticles to deliver doxorubicin for glioma therapy with success, and it demonstrated the ability of migration, infiltration, and good drug loading characteristic of the M1 phenotype, besides reflecting the strong phagocytic ability of these cells [165].

In a recent study, it was demonstrated that nanoparticle properties such as elasticity, composition, surface charge, and size influence transendothelial migration of monocytes in a human BBB model [166]. The study revealed that 200-nm-sized protein-based particles increased the migration of loaded monocytes by two-fold, whereas a much bigger poly-(methyl methacrylate) (PMMA, 500 nm in size) reduced the migration by half. These results were confirmed by the evaluation of expression of transmigration genes by RNAseq in loaded monocytes, where different leukocyte migration genes including CXCL10, VCAM1, and ITGAM were highly upregulated in both protein-based nanoparticle loaded monocytes versus PMMA-500 loaded monocytes [166]. In another recent study, Gardell et al. created engineered human monocyte-derived macrophages to secrete a bispecific T cell engager (BiTE) specific to the mutated EGFRvIII expressed by some GBM tumors. They proved that transduced human macrophages were capable to secrete a lentivirally encoded functional EGFRvIII-targeted BiTE protein capable of inducing T cell activation, proliferation, degranulation, and killing of antigen-specific GBM tumor cells [167]. Furthermore, BiTE secreting macrophages reduced early tumor burden in both subcutaneous and intracranial mouse models of GBM, a response which was enhanced using macrophages that were dual transduced to secrete both the BiTE protein and IL-12, preventing tumor growth in an aggressive GBM model [167].

Of particular interest is the observation that TAMs localize mostly to poorly vascularized hypoxic regions of tumors, which are highly resistant to conventional treatments such as chemotherapy and RT [132,168,169,170,171,172]. Therefore, TAMs may be especially useful for the treatment of tumors with significant hypoxic regions, such as GBM.

On the other hand, macrophages have also been tested as an adoptive cell therapy with chimeric antigen receptor (CAR) immunotherapy. Since macrophages can efficiently infiltrate solid tumors, they are major immune regulators and abundantly present in TME; their therapeutic effect could be beneficial for the activation of immature dendritic cells and CD8+ T cells [173,174,175]. In a recent study by Chen Chen et al., macrophages present in GBM were exploited with CAR technology for tumor recurrency post-surgery in a GBM GL261 syngeneic orthotopic mouse model [176]. CAR gene-loaded nanoparticles in a hydrogel were able to introduce GSC-targeted CAR genes into TAM nuclei after intracavity delivery to generate CAR-macrophages. The resulting CAR-macrophages were able to seek and engulf GSCs and clear residual GSCs by stimulating an adaptive antitumor immune response and preventing postoperative glioma relapse by inducing long-term antitumor immunity [176].

## 9. Conclusions

Despite the advances in GBM research, there is an emerging need for identifying reliable targets in order to improve the drastic survival rates of GBM patients. The understanding of the biological and molecular behavior of different GBM subtypes, such as specific mutations in IDH, have contributed to deciphering the prognosis of disease, and design new therapeutic opportunities. However, studies should not focus solely on the tumor cells. The GBM tumor development environment plays an essential role in the progression of the disease, in which non-tumor cells intervene, collaborating in the progression and resistance to therapies. Different tumor niches are developed into GBM tissue where TAMs represent the most abundant immune cells of the TEM contributing with molecular signaling for tumor progression and resistance to conventional therapies. Therefore, TAMs may be appropriate candidates to target or to use as cellular therapy, taking advantage of its “home to” capacity through the recruitment of monocyte precursors from bloodstream. The molecular targeting strategies to deplete or reprogram TAMs in GBM tumor niches has generated several new drugs that are at best in clinical investigation for recurrent GBM with a modest efficiency increasing OS. From the analysis of these new molecular targets, it a better performance can be appreciated when combined with other therapeutic approaches. New therapeutic challenges must focus on multiple combinations of treatments to eradicate or improve survival in patients with GBM due to molecular targeting focus in TAMs selectivity, not significantly improve GBM survival by itself. This multiple approach may come from new alternative therapies under investigation, such as photo-assisted therapies that have the advantage of being able to be combined with other treatments without adding adverse secondary effects. On the other hand, the role of these alternative treatments on the TME and specifically on the macrophage population in GBM requires further studies to determine a possible synergistic action.

## Figures and Tables

**Table 1:** Main functions of cellular components of TME of GBM.

TME Cellular Components	Functions
Astrocytes	Homeostasis regulation
Endothelial cells	Angiogenesis and BBB formation
Microglia	Immune regulation
M1-like macrophages	Proinflammatory
M2-like Macrophages	Anti-inflammatory and tumor progression promoter
Neurons	Receive, process, and transmit information
Pericytes	Angiogenesis and BBB formation
GSCs	Tumor perpetuation and resistance

## Data Availability

Not applicable.

## Abbreviations

ADAM: A Disintegrin and Metalloprotease, Ang-1: Angiopoietin-1, anti-CTLA-4 mAb: monoclonal antibody against CTL antigen 4, APC: antigen presenting cell, Arg1: arginase 1, ATMPs: advanced therapy medicinal products, ATRX: alpha-thalassemia/mental retardation, X-linked, BBB: blood brain barrier, BBTB: blood–brain tumor barrier, BEHAB: brain-enriched hyaluronan binding, BiTE: bispecific T cell engager, BMDM: bone-marrow-derived macrophages, c-Kit: tyrosine–protein kinase Kit, CAR: chimeric antigen receptor, CCL: chemokine C-C ligand 5, CCR: C-C chemokine receptor, CD: cluster of differentiation, CD62L: L-selectin, CNS: central nervous system, COX-2: cyclooxygenase 2, CSF-1R: colony-stimulating factor 1 receptor, CSF1: colony-stimulating factor 1, CSF1R: receptor of the colony-stimulating factor-1, CX3CL1: chemokine (C-X3-C motif) ligand 1, CX3CR1: CX3C motif chemokine receptor 1, Cx43: connexin43, CXCL: C-X-C motif chemokine ligand, CXCR4: chemokine receptor C-X-C type 4, DC: dendritic cell, DNA: deoxyribonucleic acid, EC: endothelial cells, ECM: extracellular matrix, EGF: epidermal growth factor, EGFR: epidermal growth factor receptor, EGFRvIII: epidermal growth factor receptor variant III, GBM: glioblastomas, GSCs: glioma stem cells, HIF-1: hypoxia-inducible factor 1, Hrh1: H1 histamine receptor, IDH: isocitrate dehydrogenase, IFN-γ: interferon-γ, IGF1: insulin-like growth factor 1, IGF1R: insulin-like growth factor receptor 1, IL: interleukin, iNOS: nitric oxide synthase, LGG: low-grade gliomas, LY6C: lymphocyte antigen 6 complex, locus C1, M-CSF: Colony stimulating factor Macrophages, M1: macrophage subtype 1, M2: macrophage subtype 2, MAPK: mitogen-activated protein kinase, MCP-1: monocyte chemotactic protein-1, MDSCs: myeloid-derived suppressor cells, miRNA: microRNA, MMP: matrix metalloproteinase, mTOR: mammalian target of rapamycin, NF-kB: nuclear factor-kappa B, NF1: neurofibromin 1, NK: natural killer, NOS: not otherwise specified, OS: overall survival, PD-1: programmed death-1, PD-L1: programmed cell death-ligand 1, PDGF: platelet derived growth factor, PDGFR: receptor platelet derived growth factor, PDT: photodynamic therapy, PFS: progression-free survival, PI3K: phosphatidylinositol-3-kinase, PS: photosensitizer, RHAMM: receptor for hyaluronan-mediated motility, RNA: ribonucleic acid, ROS: reactive oxygen species, RT: radiotherapy, SCYA2: small inducible cytokine A2, SDF-1: factor 1 derived cell stroma, SLAN: 6-sulfo LacNac, SPARC: secreted protein acidic and rich in cysteine, STAT3: signal transducer and activator of transcription 3, TAMs: tumor-associated macrophages, TCB: T-cell bispecific antibodies, TGF-α: transforming growth factor α, TGF-β: transforming growth factor β, Tie2: tyrosine-protein kinase, TIL: tumor infiltrating lymphocyte, TLRs: toll-like receptors, TME: tumor microenvironment, TMZ: temozolomide, TNF-ɑ: tumor necrosis factor ɑ, TP53: tumor protein p53, Tregs: regulatory T cells, TrLp: TriCurin, TTFields: tumor-treating fields, VCAM1: vascular cell adhesion molecule 1, VEGF-A: vascular endothelial growth factor A, VEGF: vascular endothelial growth factor, VEGFR1: vascular endothelial growth factor receptor-1, WHO: World Health Organization.
